# Cancer stem cell specificity as new targets in breast tumor treatment

**DOI:** 10.32604/or.2024.050505

**Published:** 2025-03-19

**Authors:** ZHIZHENG ZHANG, TAO LI, YUAN LI, XI WANG, HAO LIU, XINYU SHEN, ANN XU, TIANSONG XIA, BO XU

**Affiliations:** 1 Department of Breast Surgery, The First Affiliated Hospital of Nanjing Medical University, Nanjing, 210029, China; 2 Department of Pathology, Jiangsu Provincial People’s Hospital, Nanjing, 210029, China; 3 Department of Gynaecology and Obstetrics, No. 908th Hospital of Chinese PLA Joint Logistic Support Force, Nanchang, 330000, China; 4 First Clinical Medical College, Nanjing University of Chinese Medicine, Nanjing, 210023, China; 5 Department of Outpatient, Eastern Theater General Hospital, Nanjing, 210016, China; 6 Zhejiang ZhongNuoMei Biotech Limited Company, Hangzhou, 310000, China; 7 School of Chinese Medicine, Nanjing University of Chinese Medicine, Nanjing, 210023, China

**Keywords:** Breast cancer, Stem cells, Immunotherapy, Gene

## Abstract

**Background:**

Breast cancer is a prevalent malignant tumor affecting females, with treatment options including surgery, radiotherapy, chemotherapy, and endocrine therapy.

**Methods:**

This review synthesizes existing literature on breast cancer stem cells and their applications in breast cancer treatment. PubMed, Web of Science, and other relevant databases were systematically searched using keywords such as “breast cancer stem cells,” “immunotherapy,” “gene therapy,” and “cell therapy.” Studies published in English were included, and their findings were analyzed to provide insights into the characteristics and therapeutic potential of breast cancer stem cells.

**Results:**

Breast cancer stem cells exhibit unique properties that contribute to tumor initiation, progression, recurrence, and therapy resistance. Immunotherapy targeting breast cancer stem cells shows promise in overcoming these challenges, but issues such as lack of specificity and drug resistance need to be addressed.

**Conclusions:**

Breast cancer stem cells represent promising targets for innovative therapeutic strategies aimed at improving treatment outcomes in breast cancer patients.

## Introduction

Breast cancer is one of the most common malignant tumors in women, and its incidence is rising all over the world [1]. Although there are a variety of treatment methods that can be used to treat breast cancer, all of these treatment effects still have shortcomings such as tumor recurrence [2], drug resistance, and other issues [3]. Research and exploration into breast cancer treatment is therefore particularly important [4,5].

Current traditional treatments for breast cancer primarily include surgical resection, radiotherapy, and chemotherapy [6], all of which target cancer cells for destruction directly. However, the discovery of the existence of breast cancer stem cells has brought challenges to traditional treatment methods because these cells have strong self-renewal capabilities and promote drug resistance and are thus prone to tumor recurrence and metastasis [7]. Therefore, it is necessary to explore novel treatment methods that can kill and eliminate the specific characteristics of breast cancer stem cells, in order to achieve better therapeutic outcomes [8]. Stem cell therapy is one such specific treatment method, and it kills and eliminates breast cancer stem cells by utilizing their own multi-directional differentiation and self-renewal abilities [9].

With the deepening development of stem cell research, more and more studies have shown that stem cell therapy can be used as a specific treatment strategy for breast cancer stem cells [10]. At present, however, the research on stem cell therapy for breast cancer stem cells is relatively limited, and more in-depth research and exploration of its mechanism, therapeutic effect, and safety are needed [7,11]. Thus, this article aims to review the role of breast cancer stem cell specificity in the treatment of breast cancer, analyze the application prospects and existing problems of stem cell therapy in the treatment of breast cancer, and provide a reference for the future treatment of breast cancer [12].

In this review, we propose to explore the role of breast cancer stem cell specificity as a novel target in breast cancer treatment. We hypothesize that understanding and targeting the unique characteristics of breast cancer stem cells could lead to more effective therapeutic interventions and improved patient outcomes. By critically evaluating the current state of stem cell therapy in breast cancer treatment, we aim to identify potential avenues for further research and development, ultimately contributing to the advancement of personalized treatment strategies for breast cancer patients.

## Characteristics of Breast Cancer Stem Cells

Breast cancer stem cells (BCSCs) are stem cells with the abilities of self-renewal, self-repair, and multi-directional differentiation [13,14]. Compared to ordinary cancer cells, the characteristics of breast cancer stem cells include the following additional aspects, discussed below.

### Self-renewal

One of the characteristics of stem cells is their ability to self-renew. Self-renewal refers to the ability of stem cells to continuously divide and produce stem cells like themselves, thereby maintaining a stable population [15,16]. Furthermore, stem cells can also generate various types of other cells through their limited differentiation ability [17], and these other cells can participate in the tissue repair and regeneration process. However, research [18] described that breast cancer stem cells (BCSCs) from two cell lines have different proliferative capacities, which would postulate that they are not stable, and this quality would be important to elucidate in order to choose the cell line as a drug carrier or in case of replacement therapies. It has been reported that the CD44high phenotype stands out as a prominent CSC antigen, whereas the CD44high/CD24low phenotype serves as one of the key identifiers for these cells in invasive breast cancer [18]. This ability of self-renewal and differentiation gives stem cells broad application prospects in many fields, such as tissue engineering, regenerative medicine, and drug discovery [19].

Self-renewal also plays a key role in breast cancer [20]. Breast cancer stem cells can not only continually divide into stem cells but can also produce cancer cells of nonstem-cell type, thus promoting the formation, proliferation, and metastasis of tumors [21]. In this process, breast cancer stem cells show high sensitivity to a variety of signal pathways, such as Notch, Wnt, Hedgehog, and PI3K/Akt [22]. Each of these signal pathways can activate self-renewal and differentiation BCSCs, thereby maintaining and expanding tumor cell populations [23].

### Immunotherapy

The role of BCSCs in immunotherapy is an area of major focus in current research. Immunotherapy can achieve the goal of treating breast cancer by activating the patient’s own immune system in order to attack and eliminate tumor cells. However, as the “seed” of tumor cells, BCSCs also play an important role in immunotherapy [24,25]. Research showed that BCSCs have certain immune escape capabilities [26]. They can evade immune attacks by inhibiting T cell activity and inhibiting tumor cell apoptosis. In addition, BCSCs can quickly and easily proliferate and regenerate, allowing them to recover and further expand after immunotherapy [27]. Therefore, when treating breast cancer, treating BCSCs directly is of paramount importance in maximizing efficacy [28].

In the quest to effectively combat breast cancer, understanding the intricate interplay between BCSCs and the immune system is paramount. This interaction serves as a critical determinant of tumor progression and therapeutic response. Therefore, a comprehensive approach encompassing various facets is necessary to address this challenge effectively. Firstly, delving into the interaction mechanism between BCSCs and immune cells is crucial. Research must focus on elucidating how BCSCs evade immune surveillance and establish an immunosuppressive microenvironment conducive to their survival and proliferation. By deciphering these intricate molecular pathways, novel therapeutic targets can be identified to reinvigorate the immune response against BCSCs. Strategies such as immune checkpoint inhibitors, chimeric antigen receptor (CAR) T-cell therapy, and cancer vaccines hold promise in activating immune cells to specifically target and eliminate BCSCs.

BCSCs possess inherent plasticity and can regenerate tumors even after conventional treatments. Therefore, efforts should be directed toward developing targeted therapies that disrupt the self-renewal pathways of BCSCs, rendering them susceptible to eradication. Small molecules, peptides, or nucleic acid-based therapeutics targeting key regulators of BCSC self-renewal, such as Notch, Wnt, or Hedgehog signaling pathways, present potential avenues for intervention. Additionally, combination therapies incorporating conventional cytotoxic agents with BCSC-targeted agents can synergistically enhance treatment efficacy by preventing tumor recurrence [29].

Investigating the role of BCSCs within the TME is essential. The TME provides a nurturing niche for BCSCs, promoting their survival, invasion, and metastasis. Understanding the dynamic crosstalk between BCSCs and stromal components within the TME is crucial for devising therapeutic strategies to disrupt this supportive milieu. Approaches such as targeting tumor-associated macrophages, cancer-associated fibroblasts, and angiogenic factors can remodel the TME, rendering it inhospitable for BCSC proliferation and dissemination [30].

In line with these research endeavors, immunotherapy emerges as a promising modality for BCSC eradication. By harnessing the power of the immune system, immunotherapeutic interventions can unleash a robust and durable anti-tumor response. Immune checkpoint inhibitors, such as programmed cell death protein 1 (PD-1) and cytotoxic T-lymphocyte-associated protein 4 (CTLA-4) inhibitors, can unleash T-cell-mediated cytotoxicity against BCSCs, thereby impeding tumor progression. Additionally, strategies aimed at activating the apoptosis pathway specifically in BCSCs hold immense therapeutic potential, leading to their selective elimination while sparing normal cells. Furthermore, stem-cell-specific targeted therapies represent a burgeoning field in BCSC eradication. Monoclonal antibodies and small-molecule inhibitors targeting surface markers or signaling pathways enriched in BCSCs offer precision and efficacy in tumor eradication. By exploiting BCSC-specific vulnerabilities, these targeted agents can disrupt crucial survival and proliferative pathways, leading to BCSC depletion and tumor regression. Moreover, combination regimens incorporating both immunotherapy and targeted therapy hold promise in overcoming treatment resistance and achieving durable responses in breast cancer patients [31].

To begin, immunotherapy can treat breast cancer by activating T cells and attacking tumor stem cells [32]. In addition, immunotherapy can activate the apoptosis pathway for BCSCs [33]. Certain stem-cell-specific targeted therapies such as monoclonal antibodies and small-molecule targeted drugs can also kill BCSCs directly [34] and can also inhibit the regeneration and proliferation of BCSCs to a certain extent [35,36].

The role of BCSCs in the TME is also a major focus of current cancer research [37]. BCSCs can regulate the TME by secreting various cytokines and growth factors, thus promoting the growth and spread of tumors [38], and regulating the immune cells in the TME can thus help to inhibit the growth and proliferation of BCSCs [39,40]. Related targets of BCSCs are summarized in Table 1.

**Table 1:** BCSCs immunotherapy targets

Target/receptor	Immunotherapy	Mechanism	References
PD-1/PD-L1	Anti-PD-1/PD-L1 antibody	Inhibits the interaction between PD-1 and PD-L1 and enhances the ability of immune cells to attack BCSCs	[41,42]
CD47	Anti-CD47 antibody	Inhibits the activity of CD47 and enhances the phagocytosis and clearance ability of immune cells for BCSCs	[43,44]
CD44	Anti-CD44 antibody	Inhibits the activity of CD44 and affects the proliferation, migration, and invasion of BCSCs	[45,46]
HER2	Anti-HER2 antibody	Inhibits the activity of HER2 and reduces the proliferation and viability of BCSCs	[47,48]
Notch	Anti-notch antibody	Inhibits the notch signaling pathway and affects the self-renewal ability of BCSCs	[49]
Wnt	Anti-Wnt antibody	Inhibits the Wnt signaling pathway, thereby reducing the proliferation and self-renewal of BCSCs	[50]

### Gene therapy

**Figure 1:**
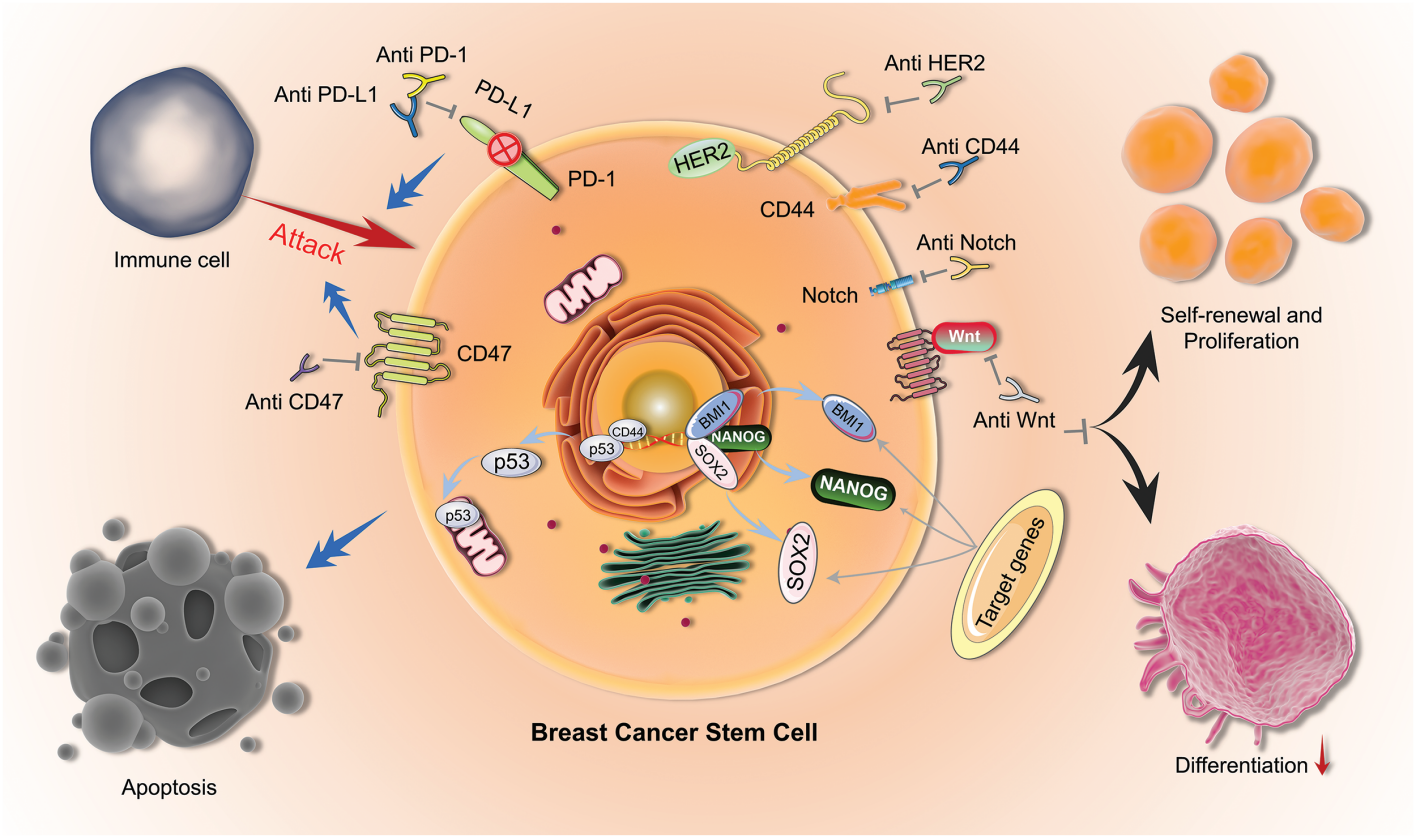
Mechanisms of BCSCs. Therapeutic mechanisms for targeting BCSCs (BCSCs) include immunotherapy using Anti-CD47, Anti-PD-1, and Anti-PD-L1; blocking self-renewal and proliferation by targeting HER2, CD44, Notch, and Wnt pathways; and inducing apoptosis by activating p53.

BCSCs are always the source of tumors. Therefore, gene therapy that targets BCSCs is an effective therapeutic strategy [51], and many genes have been found to inhibit the proliferation of BCSCs [52]. For example, the p53 gene is an important tumor suppressor gene, and its abnormal expression in breast cancer is closely related to tumor malignancy. By introducing the p53 gene into BCSCs, the proliferation of tumor stem cells can be effectively inhibited. Although gene therapy is a relatively new treatment method [53], it has already been widely discussed in the treatment of BCSCs [54]. In addition, miRNA is also an important gene therapy target. Studies have shown that miRNA plays an important role in BCSC self-renewal and proliferation, and crucially, introducing certain specific miRNAs into BCSCs has actually been found to inhibit their proliferation and differentiation [51]. Furthermore, some genes can also exert therapeutic effects on BCSCs by regulating the immune escape of tumor stem cells and enhancing tumor immune response (Table 2, Fig. 1).

**Table 2:** Genes and their functions in BCSCs gene therapy

Gene	Function	References
P53	Inhibits the proliferation and differentiation of BCSCs and increases the incidence of apoptosis	[54,55]
miRNA	Regulates the self-renewal and proliferation of BCSCs	[16,56]
CD44	Participates in the proliferation and metastasis of tumor stem cells	[46]
NANOG	Regulates the self-renewal and proliferation of tumor stem cells	[57]
BMI1	Involved in the proliferation and differentiation of tumor stem cells	[58]
SOX2	Regulates the self-renewal and proliferation of tumor stem cells	[59]

### Cell therapy

Regarding target cells, BCSCs can be susceptible to various therapeutic cells, including self antigen-specific T cells and TATA-directed T cells, along with anti-tumor NK cells [60]. Cytotoxic T lymphocytes (CTLs) and natural killer cells (NK cells) are two common types of immune cells [52,61] that play an anti-tumor role by recognizing and killing tumor cells expressing certain antigens. Modified immune cells include transgenic T cells, and chimeric antigen receptor T cells (CAR-T) [60]. However, BCSCs themselves hold promise as potential therapeutic cells for targeting breast cancer. Research indicates that BCSCs possess inherent anti-tumor properties, including the expression of various anti-tumor factors and the inhibition of growth factors, which can lead to the induction of apoptosis in tumor cells [58]. Therefore, the extraction and expansion of BCSCs, followed by the optimization of their anti-tumor potency, is a promising approach in breast cancer therapy [61]. Therefore, extracting and expanding BCSCs, concentrating their anti-tumor potency, and transplanting them into patients [62], may become an effective breast cancer treatment (Table 3).

**Table 3:** Targeted breast cancer stem cell markers and genes for cell therapy

Cell therapy methods	Targeted breast cancer stem cell markers or genes	References
CAR-T cell therapy	HER2, EGFR, MUC1, CD133	[63–65]
CRISPR/Cas9	CD44, ALDH1	[45,66,67]
ShRNA gene knockdown	SOX2, NANOG	[68]
RNA interference	CD44	[69]

## Application of BCSCs

In addition to the specific treatment of BCSCs, the application of stem cells in other breast cancer treatments also has potential value [70].

### Stem cell replacement therapy for breast cancer

Breast cancer stem cell replacement therapy is a method to treat breast cancer by using exogenous normal stem cells to replace cancer cells or abnormal stem cells *in vivo* [71]. There are two main methods of this alternative therapy: one is to transplant the patient’s own stem cells back into the body, and the other is to utilize exogenous stem cells [72]. Transplantation of a patient’s own stem cells can reduce the risk of rejection and infection, but because of the existence of BCSCs, the transplanted stem cells may also be replaced by malignant stem cells. The efficacy of this method is therefore somewhat limited. The application of exogenous stem cells can also effectively replace malignant stem cells in the body [73], but there are also risks of rejection and infection.

At present, stem cell replacement therapy for breast cancer is still in the research stage and has not been widely used in clinical practice [74]. However, with the growth in stem cell research and the continual progress of technology, we expect this method to become an effective treatment for breast cancer in the future [75].

### Using BCSCs as drug carriers

BCSCs have been considered by many researchers to be potential drug carriers. Research shows that BCSCs have the ability to locate tumor tissue selectively and release drugs, thus improving the local concentration and therapeutic effect of the drugs and reducing the damage done to normal cells.

**Figure 2:**
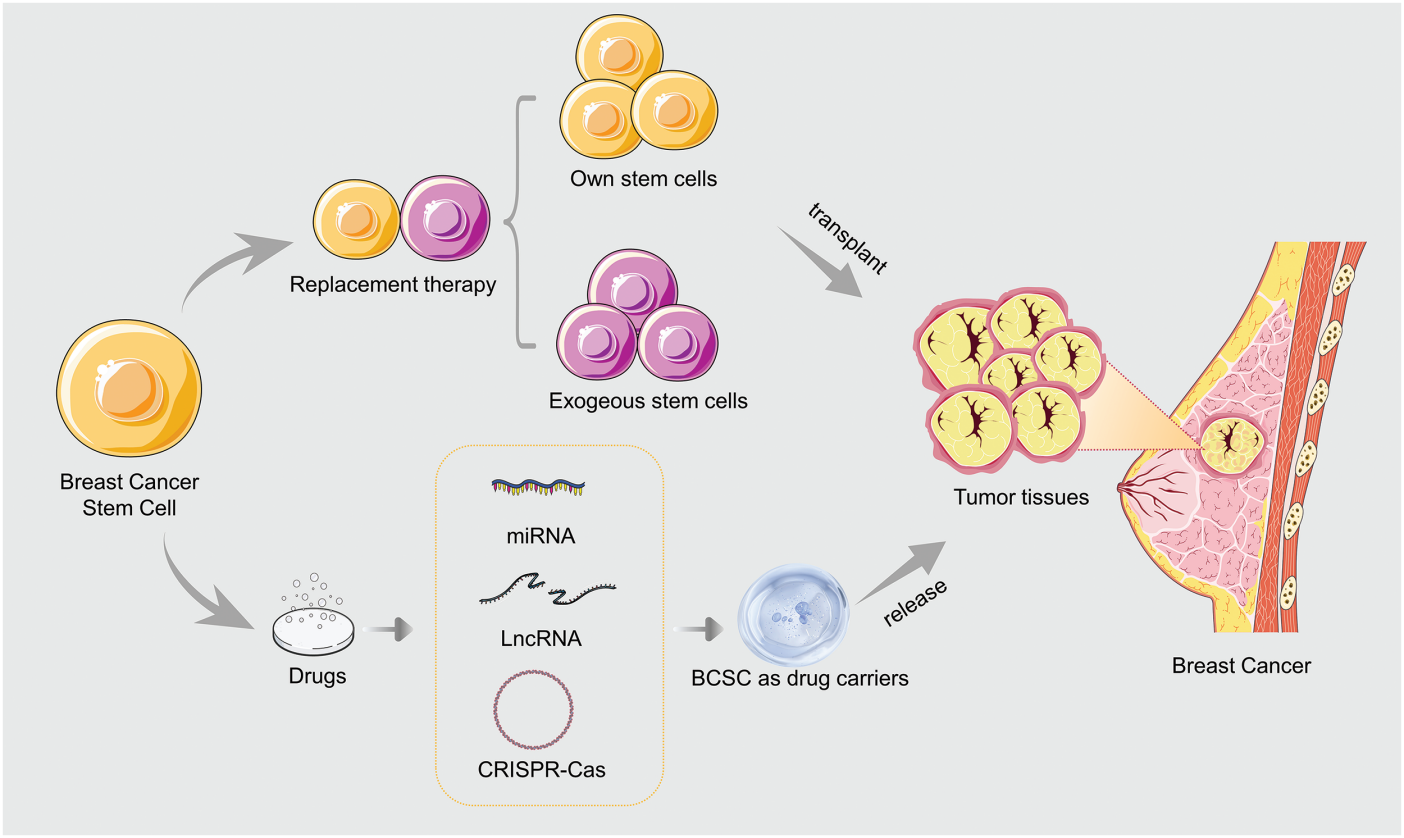
Applications of BCSCs in breast cancer treatment.

Genetic engineering technology, such as miRNAs, long noncoding RNAs (lncRNAs), and CRISPR-Cas, in which BCSCs are used as drug carriers, can transform drug-sensitive genes in BCSCs, causing them to release specific drugs. In addition, the chemical structure of drugs can be modified to interact better with BCSCs and allow for their release directly into tumor tissue [34] (Fig. 2).

## Discussion

The development of stem cell therapy in the context of breast cancer treatment represents a significant advancement in the field, offering promising avenues for targeted intervention and improved patient outcomes. Stem cell therapy, with its ability to specifically target BCSCs, holds immense potential in mitigating tumor growth and recurrence. However, the practical application of stem cell therapy is accompanied by various challenges that necessitate further technological advancements, rigorous clinical trials, and cost-effective strategies to enhance its efficacy and accessibility. One of the primary challenges in the application of stem cell therapy lies in understanding and targeting the specificity of BCSCs. BCSCs possess unique characteristics, including self-renewal capacity and resistance to conventional therapies, which contribute to tumor heterogeneity and recurrence. Therefore, developing therapeutic approaches that specifically target and eliminate BCSCs is crucial for achieving long-term remission and improving patient survival rates. DNA methylation has emerged as a key regulatory mechanism underlying BCSC properties, influencing their self-renewal, differentiation, and response to therapy. Aberrant DNA methylation patterns can lead to the dysregulation of gene expression profiles associated with BCSC phenotypes, thereby driving tumor progression and therapeutic resistance [76].

Moreover, the translation of stem cell therapy from bench to bedside faces hurdles related to technology, clinical trials, and costs. Technological advancements are required to optimize the isolation, expansion, and delivery of therapeutic stem cells, ensuring their efficacy and safety in clinical settings. Additionally, rigorous clinical trials are essential to evaluate the safety, efficacy, and long-term outcomes of stem cell-based therapies in breast cancer patients. These trials should adhere to rigorous ethical standards and regulatory guidelines to safeguard patient welfare and ensure the validity of research findings [76].

### Safety

Safety is a key issue for breast cancer stem cell therapy. Due to their self-renewal ability and differentiation potential, there are certain risks during the treatment process. Some studies have even shown that stem cell therapy may lead to tumor generation and metastasis [77]. In addition, stem cell therapy may also generate side effects such as immune responses and infections [78]. Therefore, it is necessary to monitor and control the treatment process closely, and strictly screen and identify the source, purity, and quality of stem cells. Furthermore, it is necessary to explore the optimal dosage and protocol for stem cell therapy in order to develop more effective and safe treatment methods [79].

### Effectiveness

Stem cell therapy requires selecting appropriate cell sources and effectively transferring and injecting them into the patient’s body, while also considering factors such as treatment dose and timing. However, the therapeutic effect of stem cell therapy needs to be further validated in clinical trials [80].

### Cost-effectiveness

At present, surgical treatment is still the preferred treatment for breast cancer, although with the development of stem cell biology, researchers have found that hematopoietic stem cell transplantation can play an important role in the hematopoietic and immune reconstruction of malignant tumors after high-dose radiation and chemotherapy. Moreover, mesenchymal stem cells can act as gene carriers to inhibit the growth of tumor cells. However, stem cell therapy requires a large amount of human, material, and financial investment, and its high cost so far limits its promotion and popularization in clinical applications.

Breast cancer, a prevalent malignancy in females, presents treatment challenges despite available options like surgery, radiotherapy, chemotherapy, and endocrine therapy. Limitations include post-surgery recurrence, chemotherapy’s toxicity, and side effects. Breast cancer stem cells, pivotal in cancer occurrence, recurrence, and drug resistance, offer a focal point for targeted therapies such as gene therapy, immunotherapy, and cell therapy. Immunotherapy, particularly aimed at breast cancer stem cells, garners significant research attention, albeit facing obstacles like lack of specificity, drug resistance, and safety concerns. Moreover, utilizing breast cancer stem cells as drug carriers shows promise in enhancing treatment precision and efficacy.

The limitation of this review lies in the inherent challenge of translating preclinical findings of BCSC into clinical applications, as many studies are still in the experimental stage and clinical validation is limited. Furthermore, although this review emphasizes the potential of targeting BCSCs, it does not fully address the complexity of the tumor microenvironment and its role in treatment resistance and tumor progression. The variability of BCSC markers in different patients further complicates the development of universal treatment strategies. Potential safety issues, including off-target effects and immune responses, need to be rigorously evaluated through extensive clinical trials to ensure the feasibility and effectiveness of BCSC-targeted therapy.

## Conclusion

This review highlights the potential of targeting breast cancer stem cells (BCSCs) to improve breast cancer treatment outcomes. By focusing on their unique properties, novel therapies like immunotherapy, gene therapy, and cell therapy show promise. Further research and clinical trials are essential to validate these approaches and address existing challenges.

## Data Availability

No primary data is included in this review.
